# Identification and Characterization of Auxin/IAA Biosynthesis Pathway in the Rice Blast Fungus *Magnaporthe oryzae*

**DOI:** 10.3390/jof8020208

**Published:** 2022-02-21

**Authors:** Lihong Dong, Yuming Ma, Cheng-Yen Chen, Lizheng Shen, Wenda Sun, Guobing Cui, Naweed I. Naqvi, Yi Zhen Deng

**Affiliations:** 1 State Key Laboratory for Conservation and Utilization of Subtropical Agro-Bioresources, Guangdong Province Key Laboratory of Microbial Signals and Disease Control, Integrative Microbiology Research Centre, South China Agricultural University, Guangzhou 510642, China; lihongd@stu.scau.edu.cn (L.D.); mym0736@163.com (Y.M.); slzscau@stu.scau.edu.cn (L.S.); dadasun34@gmail.com (W.S.); 20181021001@stu.scau.edu.cn (G.C.); 2 Guangdong Laboratory for Lingnan Modern Agriculture, Guangzhou 510642, China; 3 Temasek Life Sciences Laboratory, 1 Research Link, National University of Singapore, Singapore 117604, Singapore; chenchengyen1993@gmail.com (C.-Y.C.); naweed@tll.org.sg (N.I.N.)

**Keywords:** auxin, conidiation, fungus, indole-3-acetic acid (IAA), *Magnaporthe oryzae*, pathogenesis, rice blast

## Abstract

The rice blast fungus *Magnaporthe oryzae* has been known to produce the phytohormone auxin/IAA from its hyphae and conidia, but the detailed biological function and biosynthesis pathway is largely unknown. By sequence homology, we identified a complete indole-3-pyruvic acid (IPA)-based IAA biosynthesis pathway in *M. oryzae*, consisting of the tryptophan aminotransferase (MoTam1) and the indole-3-pyruvate decarboxylase (MoIpd1). In comparison to the wild type, IAA production was significantly reduced in the *motam1*Δ mutant, and further reduced in the *moipd1*Δ mutant. Correspondingly, mycelial growth, conidiation, and pathogenicity were defective in the *motam1*Δ and the *moipd1*Δ mutants to various degrees. Targeted metabolomics analysis further confirmed the presence of a functional IPA pathway, catalyzed by MoIpd1, which contributes to IAA/auxin production in *M. oryzae*. Furthermore, the well-established IAA biosynthesis inhibitor, yucasin, suppressed mycelial growth, conidiation, and pathogenicity in *M. oryzae*. Overall, this study identified an IPA-dependent IAA synthesis pathway crucial for *M. oryzae* mycelial growth and pathogenic development.

## 1. Introduction

Indole-3-acetic acid (IAA) is the active form of the phytohormone auxin, which imperatively regulates plant growth and development, as well as plant–microbe interaction [1,2,3]. Besides plants, microbes including fungi could also produce IAA, using tryptophan-dependent or -independent pathways [1,4,5]. Five tryptophan-dependent IAA synthesis pathways have been reported, namely indole-3-pyruvic acid (IPA), indole-3-acetamine (IAM), indole-3-acetonitrile (IAN), tryptamine (TPA), and the tryptophan side chain oxidase (TSO) pathway [4,6,7]. Although fungus-derived IAA has been reported, the IAA biosynthesis pathway(s) in fungi remain largely unknown. In the smut fungus *Ustilago maydis*, the IPA pathway for IAA biosynthesis was established. The enzymes involved in *U. maydis* IPA pathway were identified and characterized, including the tryptophan aminotransferases (TAMs) Tam1 and Tam2, converting tryptophan to IPA [8], and the indole-3-acetaldehyde dehydrogenases (IADs), Iad1 and Iad2, converting indole-3-acetaldehyde (IAAld) to IAA [9]. Another phytopathogenic fungus, *Leptosphaeria maculans*, uses the indole-3-pyruvate decarboxylase (IPDC) LmIPDC2 to catalyze conversion from IPA to IAAld for IAA biosynthesis [10].

The filamentous ascomycete *Magnaporthe oryzae* causes leaf and panicle blast, which is a serious disease of rice and other cereal crops. *M. oryzae* produces asexual spores, conidia, which contact the host surface and upon sensing suitable environmental signals, and differentiate into dome-shaped structures named appressoria for penetrating and infecting the host plants [11,12,13]. During rice–*M. oryzae* interaction, phytohormones produced by plant and/or fungus play an important role in the host–pathogen communication. It has been reported that accumulation of IAA in rice causes enhanced susceptibility to *M. oryzae*, while blocking rice IAA synthesis or signaling would help the rice gain resistance to blast [14]. Although *M. oryzae* was also reported to synthesize and secrete IAA [15], the detailed biosynthesis pathway and the physiological function of such fungus-derived IAA are unclear.

In this study, we identified *M. oryzae* orthologs of *U. maydis* TAMs and IADs, as well as of *L. maculans* IPDC, namely MoTam1, MoIad1, MoIad2, and MoIpd1. Reverse genetics and phenotyping analysis demonstrated that MoTam1 and MoIpd1 are involved in fungal auxin/IAA biosynthesis, and play a crucial role in fungal growth, differentiation, and pathogenesis. LC-MS analysis demonstrated that MoTam1 and MoIpd1 contributed to IAA biosynthesis in *M. oryzae*, likely via the IPA pathway. Our targeted metabolomics analysis further confirmed that the MoIpd1-mediated IPA pathway is a major contributor of IAA biosynthesis *M. oryzae*. In summary, our study reveals a fungal IAA biosynthesis pathway, and its important function in fungal pathogenicity.

## 2. Materials and Methods

### 2.1. Fungal Strains, Growth Conditions, and Genetic Transformation

The *M. oryzae* strain B157 was used as the wild type in this study. The wild type and the derived transformants/mutant strains were grown on prune agar medium (PA) or complete medium (CM) at 28 °C for 7 days before measurement of the colony sizes. For detection of IAA in *M. oryzae*, the fungal mycelia were grown in liquid complete medium with basal nitrogen (CMN) under dark condition (28 °C, 180 rpm) for 2 days. In this study, the deletion mutants were generated by *Agrobacterium tumefaciens*-mediated transformation (ATMT) of *M. oryzae* [16].

### 2.2. Blast Infection Assay

The seeds of the rice cultivar CO39 were surface sterilized and allowed to germinate on wet filter paper, incubated at 24 °C, humidity > 90%, for 2–3 days. The germinated seeds were planted in the pots with soil. Cultivation of the barley seedlings followed the similar methodology. Leaf blades of the 2-week-old rice or barley seedlings were used for blast infection assays. The freshly harvested conidia, or the fungal mycelial plugs from 7-day-old cultures, were used for infecting the detached rice or barley leaves. The inoculated leaf explants were kept in a growth chamber with settings of 80% humidity, 28 °C, and a 12 h:12 h day:night cycle. Blast disease symptoms were examined and imaged at 7 dpi.

### 2.3. Chemical Reagents Used in This Study

Tryptophan (Trp, Sigma-Aldrich, St. Louis, MO, USA) was dissolved in water to 10 mg/mL as a stock. Auxin (IAA; Sigma-Aldrich, St. Louis, MO, USA): stock concentration of 50 mM in ethanol. 5-(4-chlorophenyl)-4H-1, 2, 4-triazole-3-thiol (yucasin; Sigma–Aldrich, St. Louis, MO, USA): stock concentration of 0.5 M in DMSO.

### 2.4. Plasmid Constructs and Fungal Transformants

The deletion constructs pKO-TAM1, pKO-ARO8, pKO-ARO9, pKO-ARO98, pKO-IAD1, and pKO-IAD2 were individually generated based on the plasmid pFGL821 (with the hygromycin resistance gene, *HPH*) or pFGL822 (with the glufosinate ammonium resistance gene, *BAR*) by surrounding the resistant gene (selection marker) with the homologous regions of the targeting gene, respectively. The deletion constructs were individually transformed into the wild-type strain to generate the corresponding deletion mutants. For generating the *moaro9*Δ *moaro8*Δ double deletion mutant, the pKO-ARO98 was transformed into the *moaro8*Δ mutant. The primer sequences used for generating the deletion constructs are listed in Table S1. The generation and characterization of the *moipd1*Δ mutant were reported in our previous preprint [17].

### 2.5. Nucleus Acid Manipulation

Genomic DNA extraction from mycelia was performed following the SDS protocol [18]. Southern blot analysis was performed following an established protocol [19].

### 2.6. Tryptophan Metabolomics Analysis

Sample preparation: The fungal mycelia were cultured in CMN liquid medium with/without tryptophan (1 mg/mL) for 2 days, then weighed (100 ± 5 mg per sample) and homogenized using Biospec MiniBeadbeater24 (Fastprep24). A 10 μL internal standard (IS: containing L-Kynurenine-d4, 2-Picolinic-d4 Acid, 5-Hydroxyindole-3-acetic-2,2-d2 Acid, 3-Hydroxyanthranilic Acid-d3, Tryptamine-d4 Hydrochloride, Melatonin-d4, 3-Indoxyl Sulfate-d4 Potassium Salt, Serotonin-d4 Hydrochloride, and Indole-d7 of respective optimal concentrations) solution and 0.5 mL water–acetonitrile–methanol (1:2:2, *v*/*v*/*v*) solution were added into the homogenate. About 400 μL of supernatant from the sample was collected and dried under nitrogen gas after centrifugation. The residue was re-dissolved in 100 μL acetonitrile–water (1:1, *v*/*v*) solution and then centrifuged at 14,000× *g*. The supernatant was injected for HPLC-MS/MS analysis.

HPLC-MS/MS analysis: The separation was performed on a UPLC system (Agilent 1290 Infinity UHPLC) using a C-18 column (Waters, CSH C18 1.7 μm, 2.1 mm × 100 mm column) by gradient elution. Eluent A and B were acetonitrile and water consisting of 20 mM ammonium formate buffer (pH = 3.7), respectively. The gradient elution program was as follows: 0 min: 15% B, 2 min: 15% B, 9 min: 98% B, 11 min: 98% B, 11.5 min: 15% B, and 14 min: 15% B. Before injecting the next sample, the column was equilibrated with the initial mobile phase for 5 min. The flow rate was at 0.4 mL/min and the column temperature was set at 50 °C.

The 5500 QTRAP (AB SCIEX) was performed in positive and negative switch mode. The ESI positive source conditions were as follows: source temperature: 550 °C, ion source Gas1 (Gas1): 55, ion source Gas2 (Gas2): 55, curtain gas (CUR): 40, and ion sapary voltage floating (ISVF): +4500 V. The ESI negative source conditions were as follows: source temperature: 550 °C; ion source Gas1 (Gas1): 55, ion source Gas2 (Gas2): 55, curtain gas (CUR): 40,and ion sapary voltage floating (ISVF): −4500 V. The MRM method was used for mass spectrometry quantitative data acquisition. The APCI source conditions were as follows: source temperature: 550 °C, ion source Gas1 (Gas1): 55, ion source Gas2 (Gas2): 55, curtain gas (CUR): 40, and ion sapary voltage floating (ISVF): +5500 V. The MRM method was used for mass spectrometry quantitative data acquisition.

### 2.7. Extraction and Detection of IAA by LC-MS

Sample preparation and detection of intracellular or secreted IAA from liquid-cultured mycelia of the wild-type or mutant strains follows our previous optimal protocols [17]. IAA (Sigma, cat# I2886) at 200 ppb served as a standard control.

### 2.8. Data Analysis

Qualitative Analysis (version B.06.00), MultiQuant (version 3.02), or Analyst (version TF1.17) software was used for quantitative data processing. For statistical analysis, the one-way analysis of variance (ANOVA) test was carried out (*p* < 0.05, significant).

## 3. Results

### 3.1. Identification of Tryptophan-Dependent IAA Synthesis Pathway in M. oryzae

**Figure 1:**
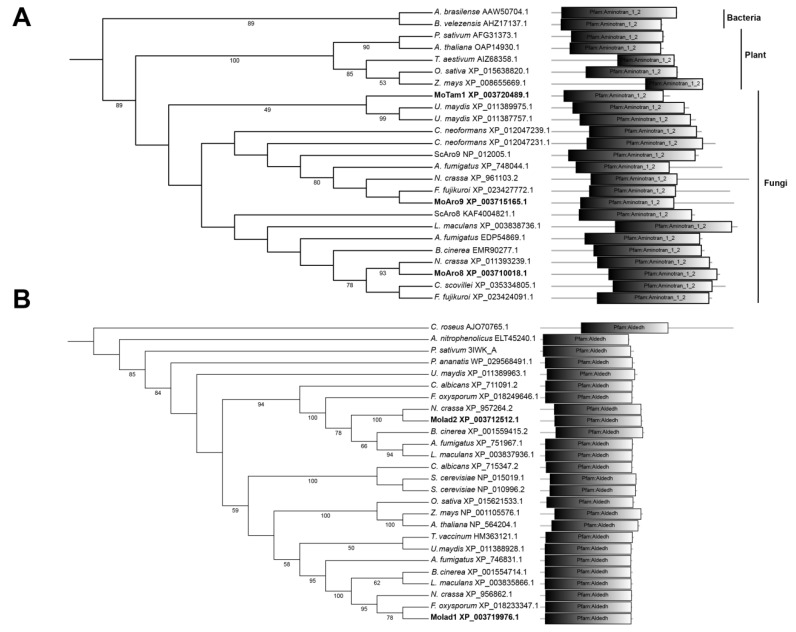
Phylogenetic analysis of TAM and IAD orthologs. (**A**) TAM orthologs; (**B**) IAD orthologs. The evolutionary history was inferred using the neighbor-joining method [30]. The percentage of replicate trees in which the associated taxa clustered together in the bootstrap test (1000 replicates) are shown next to the branches [31]. Branches corresponding to partitions reproduced in less than 50% bootstrap replicates were collapsed. The evolutionary distances were computed using the Poisson correction method [32] and in the units of the number of amino acid substitutions per sites. Evolutionary analyses were conducted in MEGA7 [33]. Domain annotation was performed using Smart website (http://smart.embl.de/, accessed on 13 December 2021).

In order to identify the possible IAA biosynthesis pathway(s) in *M. oryzae*, we searched the *M. oryzae* genome for the orthologous enzymes of the reported tryptophan-dependent pathways, using the BLAST website (https://blast.ncbi.nlm.nih.gov/Blast.cgi, accessed on 10 December 2021). The result shows that intact TPA, IPA, IAM, and TSO cascades are present, but some component in IAN pathways is missing (Table 1). Particularly, orthologs of the characterized TAM, IPDC, and IAD enzymes of the IPA pathway established in phytopathogenic fungi [8,9,10] were identified (Table 1), indicating a functional IPA pathway responsible for IAA production in *M. oryzae*. We further performed phylogenetic analysis using these TAM, IPDC, and IAD orthologs in yeast and fungi. As shown in Figure 1A, *M. oryzae* MGG_14221 (XP_003720489.1) is close to *U. maydis* Tam1 (XP_011387757.1) and Tam2 (XP_011389975.1), while MGG_09919 (XP_003710018.1) and MGG_08189 (XP_003715165.1) are in another clade with *Saccharomyces cerevisiae* Aro8 (KAF400482.1) and Aro9 (NP_012005.1), which are involved in Ehrlich pathway for production of aromatic alcohols [20]. Therefore, we named MGG_14221 as MoTam1, and MGG_09919 and MGG_08189 as MoAro8 and MoAro9, respectively. There is only one ortholog of IPDC, MGG_01892, found in *M. oryzae*, and is closer to ascomycetous fungi rather than yeast or basidiomycetous fungi [17]; therefore, we named it as MoIpd1. MGG_03900 (XP_003719976.1) and MGG_05008 (XP_003712512.1) are close to *U. maydis* Iad1 (XP_011388928.1) and Iad2 (XP_011389963.1), respectively (Figure 1B), and therefore named as MoIad1 and MoIad2, respectively.

**Table 1:** Identification of *M. oryzae* orthologs in tryptophan-dependent IAA biosynthesis pathways.

Enzyme [Reference]	*M. oryzae* ID	Bait	Identity (%)	E Value
TPA pathway				
TDC [21]	MGG_03869	CAA47898.1	25.63	8 × 10^−24^
AOX [5]	MGG_10751	P49250.1	30.63	7 × 10^-78^
IPA pathway				
TAM [8]	MGG_14221	XP_011387757.1	39.81	4 × 10^−98^
	MGG_08189		24.91	4 × 10^−31^
	MGG_09919		27.20	1 × 10^−47^
IPDC [10]	MGG_01892	XP_003844157.1	30.00	4 × 10^−70^
IAD [9]	MGG_03900	XP_011388928.1	34.18	1 × 10^−90^
	MGG_05008	XP_011389876.1	34.02	2 × 10^−88^
YUCs [22]	MGG_07629	NP_194980.1 (YUC1)	27.00	4 × 10^−32^
		NP_193062.1 (YUC2)	25.07	5 × 10^−24^
		NP_171955.1 (YUC3)	29.53	2 × 10^−31^
		NP_196693.1 (YUC4)	28.15	7 × 10^−30^
		NP_199202.1 (YUC5)	26.10	5 × 10^−28^
		NP_197944.2 (YUC6)	25.15	2 × 10^−22^
		NP_180881.1 (YUC7)	27.41	1 × 10^−27^
		NP_194601.1 (YUC8)	27.78	4 × 10^−31^
		NP_171914.1 (YUC9)	28.82	9 × 10^−32^
	MGG_04751	NP_175321.1 (YUC10)	25.49	6 × 10^−14^
	MGG_07629	NP_173564.1 (YUC11)	28.82	1 × 10^−30^
IAM pathway				
TR2M [23]	MGG_16373	AAD30489.1	39.70	1 × 10^−6^
IAM hydrolase [24]	MGG_15383	AEX60887.1	32.07	7 × 10^−63^
IAN pathway				
TPH [25]	MGG_03352	OAP11515.1	25.05	6 × 10^−30^
IAOx dehydratase [26]	MGG_14859	OAP11700	24.36	2 × 10^−27^
Nhase [27]	No hit	AFY70185.1		
NIT [28]	MGG_03280	KIS68359.1	33.75	7 × 10^−53^
TSO pathway				
TSO [29]	MGG_16230	SCZ74856.1	38.68	4 × 10^−97^

**Figure 2:**
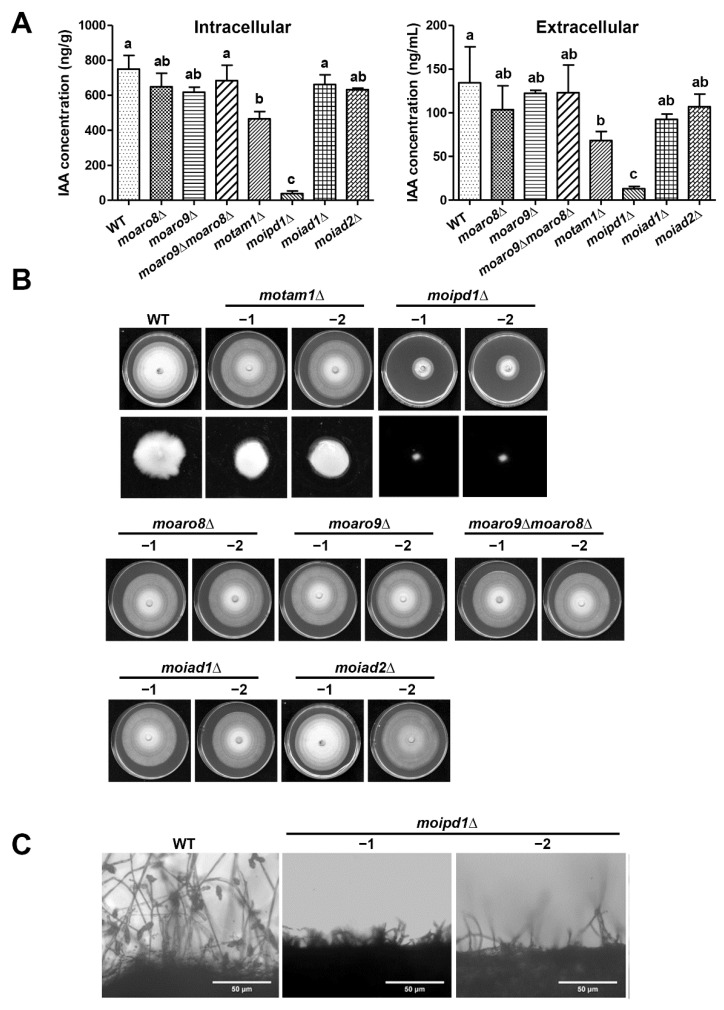
Characterization of the mutants in IAA biosynthesis, growth, and conidiation. (**A**) The mycelia of wild-type (WT) strain and mutants were grown in liquid CMN supplemented with 1 mg/mL tryptophan for 2 days, and subject to detection of IAA extracted from fungal mycelia (**left** panel) or the culture filtrate/supernatant (**right** panel), respectively. Mean ± S.E. were derived from three independent biological repeats. Different letter code represents significant difference (*p* < 0.05). (**B**) Colony morphology of the wild-type strain (WT), and the mutants grown on CM plate at 28 °C. Photographs were taken at 7 dpi. The low panel showed mycelial fluff of WT, *motam1*∆, and *moipd1*∆, grown in liquid CM for 2 days. (**C**) Mycelial culture of WT and the *moipd1*Δ mutants was exposed to light for 24 h to induce conidiation and assessed by microscopy. Scale bar = 50 μm.

To verify whether this potential IPA pathway contributes to IAA production, we generated the single deletion mutant of each gene (Figures S1 and S2) and measured the IAA content. We found that IAA content was significantly reduced in the *moipd1*Δ mutant (Figure 2A), consistent with our previous findings [17]. A reduced IAA level was detected in the *motam1*Δ mutant as compared to the wild type, but was relatively higher than that in the *moipd1*Δ mutant (Figure 2A). On the other hand, no obvious defect of IAA production was found in the *moaro8*Δ or *moaro9*Δ mutant (Figure 2A). Furthermore, the *moaro9*Δ*moaro8*Δ double deletion mutant was generated (Figure S1C), and the IAA content also did not change much in the double deletion mutant (Figure 2A). We conclude that MoTam1 and MoIpd1 are involved in tryptophan-dependent IAA production, likely via the IPA pathway, while MoAro8 and MoAro9 are not. On the other hand, the *moiad1*Δ and *moiad2*Δ mutants were also able to produce IAA to a comparable level as the wild type (Figure 2A). We infer that they are not authentic IAD enzymes in *M. oryzae*; alternatively, they are functionally redundant with each other.

### 3.2. MoTam1 and MoIpd1 Regulate Mycelial Growth, Conidiation, and Pathogenicity of M. oryzae

We next investigated the function of IAA biosynthesis in *M. oryzae*, by characterizing mycelial growth, conidiation, and pathogenicity using the aforementioned mutants. The *motam1*Δ and *moipd1*Δ mutants showed an obvious defect in mycelial growth under the liquid culture condition, while only the *moipd1*Δ mutant was defective in growth on solid medium (Figure 2B). On the other hand, the *moaro8*Δ, *moaro9*Δ, and the *moaro9*Δ*moaro8*Δ double mutant were comparable to the wild-type strain in mycelial growth (Figure 2B). The *moiad1*Δ and *moiad2*Δ mutants showed no defect in mycelial growth (Figure 2B). We quantified conidia produced by the wild-type or mutant strains and found that the *moipd1*Δ mutant was unable to produce any conidia (Figure 2C; Table 2). Except for the *moipd1*Δ mutant, all the other mutants produced conidia to a comparable level as the wild type (Table 2).

**Figure 3:**
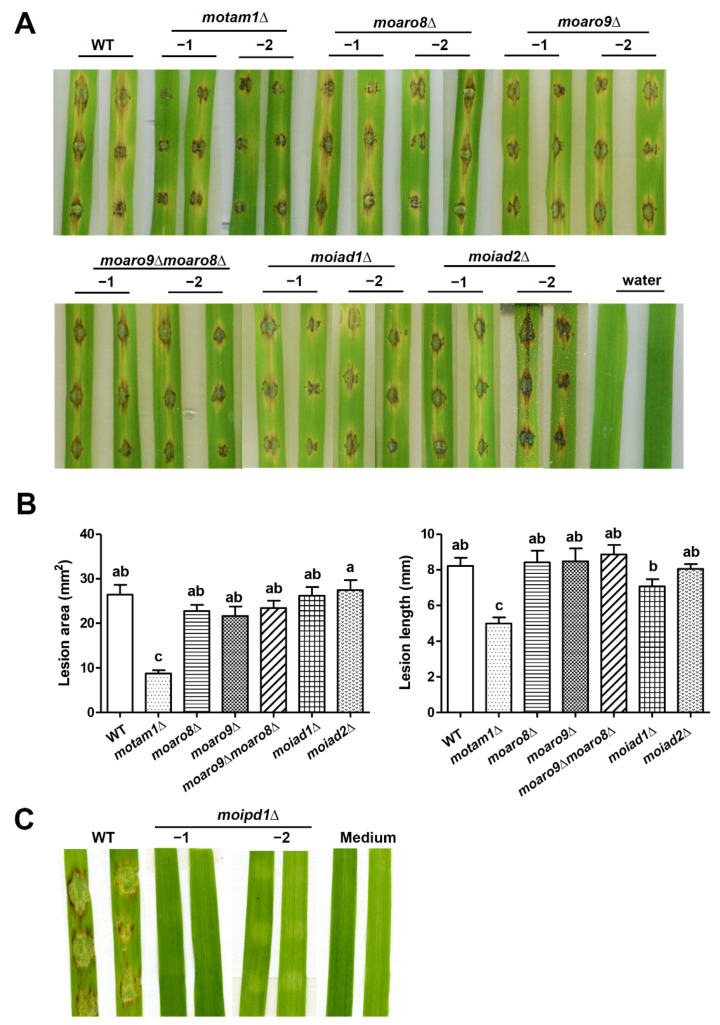
Pathogenicity assay. Fresh conidial suspension of the wild-type (WT) strain or indicated mutants was inoculated on 2-week-old rice leaf explants, respectively. Rice leaf explants inoculated with water served as blank control. The inoculum of conidia was 2000 conidia per droplet. Blast disease symptoms were (**A**) imaged and (**B**) quantified based on lesion area (**left** panel) and lesion length (**right** panel), respectively, at 7 dpi. Different letters in (**B**) denote statistically significant difference (*p* < 0.05). (**C**) The mycelial plugs of the wild-type (WT) or *moipd1*Δ mutants were inoculated on the barley leaf explants. Fresh medium plugs served as the blank control. Photos were taken at 7 dpi. For all the pathogenicity assays, three independent repeats were performed, and the representative images are displayed here.

We tested the pathogenicity of all the aforementioned mutants, except for *moipd1*∆, by inoculating the conidial suspension to the rice leaves. The *motam1*Δ mutant showed reduced pathogenicity (Figure 3A,B), while the *moaro8*Δ, *moaro9*Δ, *moaro9*Δ *moaro8*Δ double mutant, and the *moiad1*Δ and *moiad2*Δ mutants displayed comparable pathogenicity to the wild type (Figure 3A,B). As for the *moipd1*∆ mutant, which produced no conidia, we tested its pathogenicity by inoculating the mycelial plugs on the barley leaf explants. The result showed that the *moipd1*∆ mycelia are unable to cause blast disease lesions on the barley leaves (Figure 3C).

Overall, the mutants defective in IAA production (*motam1*Δ and *moipd1*Δ) were defective in mycelial growth, asexual differentiation, and pathogenicity, to various degrees, indicating that IAA biosynthesis mediated by the MoTam1-MoIpd1 pathway, likely the IPA pathway, is crucial for *M. oryzae* growth, differentiation, and pathogenicity.

### 3.3. The IPA Pathway Is A Major Contributor of IAA Biosynthesis in M. oryzae

**Figure 4:**
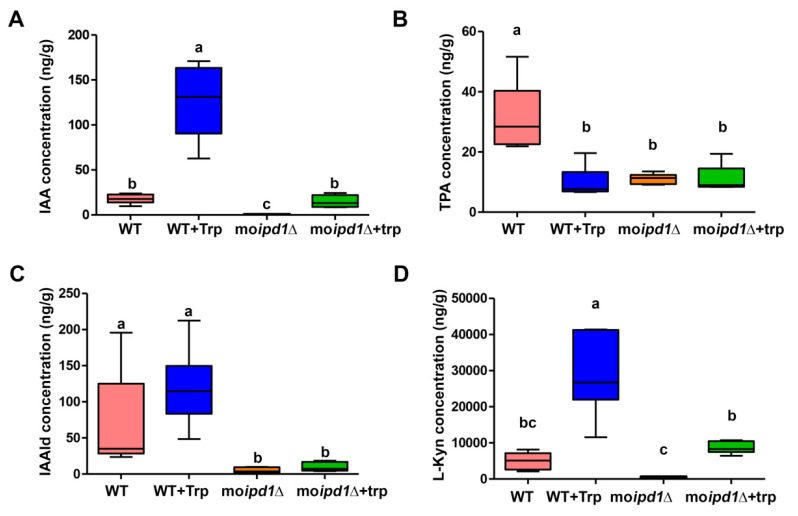
MoIpd1 is crucial for IAA biosynthesis of *M. oryzae*. The wild-type (WT) strain and *moipd1*∆ mutant were grown in CMN liquid medium with/without 1 mg/mL tryptophan (Trp) under dark conditions for 2 days. Metabolites including (**A**) IAA, (**B**) TPA, (**C**) IAAld, and (**D**) L-Kyn were detected by targeted metabolomics and plotted. Different letters denote statistically significant difference (*p* < 0.05).

As the *moipd1*Δ mutant displayed the strongest phenotypes in IAA production and pathogenic development of *M. oryzae*, we further investigated its role in IAA biosynthesis by comparative metabolomics analysis using the wild-type and the *moipd1*Δ mycelia, with or without supplementation of tryptophan. Our results (Supplementary Dataset S1; Figure 4A) showed that IAA content in the wild-type mycelia was significantly increased when tryptophan was added, further confirming that *M. oryzae* indeed uses the tryptophan-dependent pathway(s) for IAA biosynthesis. Deletion of *MoIPD1* resulted in significantly reduced IAA level in comparison to the wild-type strain (Supplementary Dataset S1; Figure 4A), confirming that the IPA pathway mediated by the IPDC enzyme MoIpd1 exists in *M. oryzae*, and is the major contributor for IAA synthesis. We noticed that IAA level increased with external supplementation of tryptophan to the *moipd1*Δ mycelia (Supplementary Dataset S1; Figure 4A), suggesting other tryptophan-dependent IAA biosynthesis pathway(s) may also exist in *M. oryzae*.

The presence of TPA (Supplementary Dataset S1) supports the existence of TPA pathway for IAA biosynthesis in *M. oryzae*. We noticed that TPA content was reduced in the wild type upon addition of tryptophan, while it remained unchanged in the *moipd1*Δ mutant (Supplementary Dataset S1; Figure 4B). On the other hand, IAAld, a common downstream metabolite of IPA, TPA, and TSO pathways [34,35], was also detected in both the wild-type strain and the *moipd1*Δ mutant (Supplementary Dataset S1; Figure 4C). We assume that IAAld of the *moipd1*Δ mutant may be sourced from TPA conversion, or directly from tryptophan catalyzed by the TSO enzyme. IAAld was significantly reduced in the *moipd1*Δ mutant as compared to the wild type (Figure 4C), indicating that neither TPA nor TSO pathway are a major contributor of IAA biosynthesis in *M. oryzae*.

Besides IAA biosynthesis, tryptophan could also be catabolized by kynurenine pathway, producing L-kynurenine (L-Kyn) as one intermediate metabolite [36], and as an inhibitor of IAA biosynthesis [37]. The L-Kyn content was significantly induced in the wild type upon addition of tryptophan (Figure 4D), indicative of a negative feedback regulation of tryptophan-dependent IAA biosynthesis. L-Kyn was also induced in the *moipd1*Δ mutant, albeit to a lesser extent (Figure 4D). Overall, our results identified at least two IAA synthesis pathways, namely IPA and TPA, in *M. oryzae*, and also uncovered the kynurenine pathway for tryptophan catabolism and potential regulation of IAA biosynthesis.

### 3.4. YUCCA Pathway May Also Contribute to M. oryzae Growth, Conidiation, and Pathogenesis

**Figure 5:**
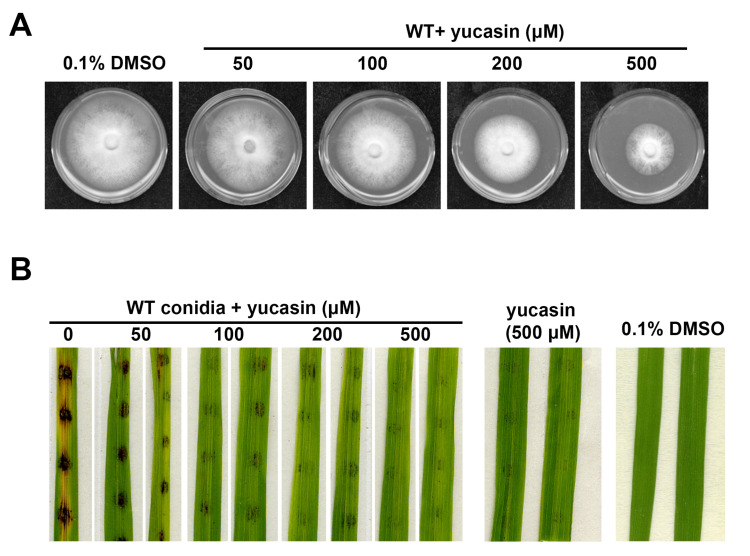
IAA biosynthesis inhibitor suppressed *M. oryzae* growth and pathogenesis. (**A**) Wild-type (WT) strain was grown on PA medium supplemented with yucasin of different concentrations, kept in dark under 28 °C. Photographs were taken at 7 dpi. PA medium containing 0.1% DMSO was set as solvent control. (**B**) Yucasin was added to the wild-type (WT) conidial suspension (2000/droplet) to reach the indicated final concentration. Photos were taken at 7 dpi. Rice leaf explants inoculated with yucasin solution (500 μM) served as a blank/mock control, and 0.1% DMSO as a solvent control.

We noticed that there are two orthologs of YUCCA (YUC; Table 1), which was reported to catalyze the conversion from IPA to IAA directly [38], thus suggesting that the YUC pathway for auxin/IAA synthesis may also function in *M. oryzae*. Thus far, the YUC pathway has only been reported in plants [22]. To test the potential function of YUC pathway, we applied yucasin, an inhibitor of IAA biosynthesis that suppresses YUC enzymes, to the mycelial culture of *M. oryzae*. We found that yucasin could effectively suppress *M. oryzae* mycelial growth (Figure 5A; Table 1) and conidiation (Table 1). Furthermore, yucasin-treated wild-type conidia displayed obvious reduction in blast lesion development on rice leaf explants (Figure 5B), thus indicating that IAA biosynthesis via the YUC pathway plays an important role during *M. oryzae* pathogenicity. We noticed that yucasin treatment in the absence of conidia caused hypersensitive response (HR) or disease resistance-like symptoms on the rice leaf explants (Figure 5B), possibly reflecting the consequence of the inhibitory effect of yucasin on rice auxin synthesis. Overall, our results suggest that the IPA-based biosynthesis of IAA, either via MoIpd1-catalyzed IPA to IAAld conversion or via YUCs-catalyzed IPA to IAA conversion, plays a crucial role in *M. oryzae* growth, asexual development, and pathogenicity.

## 4. Discussion

Auxin (IAA) is an important phytohormone regulating cell growth and differentiation in plants [4]. Accumulating evidence demonstrates that microbes including bacteria and fungi could also synthesize and secrete auxin/IAA [5,39], but the physiological function of such microbe-sourced auxin/IAA remains largely unknown. As the rice blast causal fungus, *M. oryzae* was reported to be able to produce IAA from its hypha and conidia [15], yet the IAA biosynthesis pathway is unclear. By searching the *M. oryzae* genome for the orthologs of the enzymes of the five tryptophan-dependent pathways, we found components of TPA, IPA, IAM, and TSO pathways, but some components in the IAN pathway are missing (Table 1). Interestingly, we identified two potential YUC enzymes in *M. oryzae* orthologs of the 11 established *Arabidopsis thaliana* YUCs (Table 1). So far, the YUC pathway for auxin/IAA synthesis has only been reported in plants [22], and not in bacteria or fungi. We deduce that *M. oryzae* may also synthesize IAA directly from IPA using such YUCs. Supporting this hypothesis, the IAA inhibitor yucasin that targets YUCs [38] could also suppress mycelial growth, conidiation, and pathogenicity in *M. oryzae* (Figure 5; Table 2). We reported similar phenotypes caused by treatment of amino-oxyacetic acid (AOA) with *M. oryzae* [17], which is known to inhibit IAA biosynthesis by targeting TAM, albeit at a much lower efficiency [40]. Furthermore, suppression of IAA biosynthesis via gene-deletion analysis of the IPA pathway led to reduced growth to varying degrees under in vitro culture conditions (Figure 2B,C; Table 2), indicating that IAA plays a positive role in fungal growth. When infecting host rice, IAA mutants (*motam1*∆ and *moipd1*∆) displayed reduced or complete loss of pathogenesis (Figure 3A–C), which may be due to reduced fungal invasive growth. Alternatively, fungus-derived auxin/IAA may function in suppressing immunity in the host plants.

It has been reported that auxin is involved in plant responses to a wide range of biotic and abiotic stresses. In some cases, auxin was reported to positively regulate plant immunity against pathogens. For example, an elevated auxin/IAA level promotes resistance against *Rhizoctonia solani*, a fungal pathogen causing sheath blight in rice [41]. Infection by rice dwarf virus (RDV) triggers increased auxin biosynthesis and accumulation in rice, thereby activating auxin signaling to enhance rice defense against RDV infection [42]. However, in most cases, auxin is generally considered as a negative regulator of innate immunity in rice [43]. Auxin could not only suppress plant immunity by suppression of salicylic acid (SA)-mediated host defense, but also directly induces expression of virulence genes in the bacterial pathogen [44]. In cassava (*Manihot esculenta*), the auxin signaling pathway mediated by MeAux/IAAs negatively regulates the plant resistance to the bacterial pathogen *Xanthomonas axonopodis pv manihotis* (Xam) by affecting the expression of pathogenesis-related genes (MePRs), accumulation of reactive oxygen species, and callose deposition during the plant defense response [45]. Repressing auxin signaling by overexpressing indole-3-acetic acid-amido synthetase *GH3* could effectively enhance plant resistance, as reported in rice against *M. oryzae* [46], and in citrus against *Xanthomonas citri subsp. citri* (Xcc) [47]. Similarly, microRNA-mediated suppression of auxin responsive factor (ARF) genes leads to hyper-susceptibility of rice to *M. oryzae* [48]. Therefore, we could expect that elevated auxin/IAA, either derived from plant or fungal side, would facilitate *M. oryzae* infection in rice. We observed a dynamic accumulation of IAA in developing conidia, appressoria, and invasive hyphae of *M. oryzae*, and that IAA is also secreted out from conidia and is perceived by the host plant [17], thus suggesting a complex and crucial role for IAA during fungus–plant interactions.

**Figure 6:**
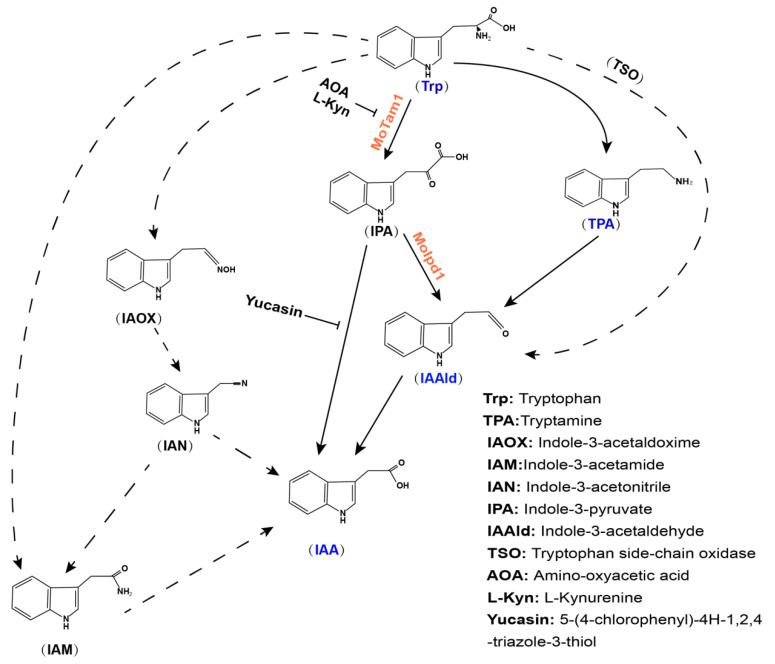
Proposed IAA biosynthesis pathway in *M. oryzae* based on tryptophan metabolomics analysis and orthologs search. Presence of pathways was inferred based on identification of *M. oryzae* orthologs (Table 1), and detection of the intermediate metabolites (Supplementary Dataset S1). Detection of tryptophan (Trp), TPA, IAAld, and IAA (blue font) indicates that the IPA and TPA pathways (solid arrows) may exist in *M. oryzae*. MoTam1 and MoIpd1 (red font) are the enzymes catalyzing IPA pathways characterized in this study. IAOX, IAN, and IAM (black font) were not assessed, so we could not conclude whether the corresponding biosynthesis pathways (dashed arrows) exist in *M. oryzae*. It is also unclear whether the TSO pathway exists in *M. oryzae*. AOA, L-Kyn, and yucasin are reported inhibitors of auxin biosynthesis.

Comparative metabolomics analysis showed that loss of ***MoIPD1*** resulted in significant reduction in IAA content, supporting that the IPA pathway is a major pathway for IAA biosynthesis in *M. oryzae*. We identified TPA and IAAld (Supplementary Dataset S1; Figure 4) in *M. oryzae*, supporting the presence of TPA pathway in *M. oryzae*. A low level of IAA was detected in the *moipd1*Δ mutant, indicating that besides the IPA pathway mediated by TAM-IPDC-IAD enzymes, other pathway(s) could also contribute to IAA biosynthesis in *M. oryzae*, likely including the YUC pathway downstream of IPA, as well as the TPA pathway that bypasses IPDC function. We could not rule out the existence of the IAN or IAM pathway in *M. oryzae*, as the corresponding intermediate metabolites were not assessed. The *M. oryzae* ortholog of the TSO enzyme could also be identified based on sequence similarity, but its biochemical activity and biological function await elucidation in the near future. Lastly, starting with the in silico search (Table 1), and followed by functional characterization of candidate genes and the relevant metabolomics analyses, we summarize the framework for tryptophan-dependent IAA biosynthesis pathways in *M. oryzae* (Figure 6).

Deletion of *MoTAM1* caused a reduction in IAA content and a mild defect in mycelial growth and pathogenicity, likely due to functional redundancy with MoAro8 and/or MoAro9, whose yeast orthologs were reported in catalyzing deamination of aromatic amino acids, including tryptophan, but for the production of aromatic alcohols [20]. It remains to be investigated whether *M. oryzae* also produce aromatic alcohols. The *moipd1*Δ mutant showed the strongest phenotype in IAA production among all the tested mutants, and correspondingly had the most obvious defects in growth and pathogenic development. We were intrigued to notice that the conidiation or pathogenicity defect of the *moipd1*Δ mutant could not be restored by exogenously supplying IAA (data not shown). It has been reported that photo-oxidation of IAA forms toxic products 3-hydroxymethyl oxindole (HMO) and 3-methylene oxindole (Meox) [49,50,51], which may suppress fungal growth. It would be of great interests to verify whether these two substances are present in an IAA-supplemented medium.

In summary, our study established a major pathway contributing to IAA biosynthesis in *M. oryzae*, catalyzed by the identified MoTam1 and MoIpd1 enzymes. The fungus-derived IAA plays an important role in regulating *M. oryzae* growth and pathogenic development, and likely mediates the interaction between fungal pathogens and plant hosts.

## Figures and Tables

**Table 2:** Comparison of mycological characteristics among strains.

Strain	Radius of Colony (Average ± S.E.)	Conidia Count (×10^5^/mL; Average ± S.E.)
Wild type (WT)	3.52 ± 0.02 ^A^	58.76 ± 5.08 ^a^
*motam1*Δ	3.53 ± 0.07 ^A^	60.58 ± 4.10 ^a^
*moaro*8Δ	3.58 ± 0.02 ^A^	65.05 ± 1.89 ^a^
*moaro9*Δ	3.50 ± 0.02 ^A^	63.25 ± 7.76 ^a^
*moaro9*Δ *moaro8*Δ	3.60 ± 0.04 ^A^	59.20 ± 6.71 ^a^
*moipd1*Δ	1.42 ± 0.03 ^D^	0 ^c^
*moiad1*Δ	3.57 ± 0.03 ^A^	60.32 ± 6.35 ^a^
*moiad2*Δ	3.52 ± 0.05 ^A^	61 ± 5.10 ^a^
WT + Yucasin (50 μM)	3.42 ± 0.03 ^A^	17.42 ± 4.49 ^b^
(100 μM)	3.05 ± 0.02 ^B^	13.55 ± 2.37 ^b^
(200 μM)	2.63 ± 0.03 ^C^	8.71 ± 2.37 ^bc^
(500 μM)	1.72 ± 0.07 ^D^	6.78 ± 1.19 ^bc^

Difference letters denote statistical significance (*p* < 0.05); *n* > 12 at each instance, of three independent biological repeats.

## Data Availability

The data that support the findings of this study are available from the corresponding author upon reasonable request.

## Supplementary Materials

The following are available online at https://www.mdpi.com/article/10.3390/jof8020208/s1, Figure S1: Construction and verification of deletion mutants; Figure S2: Construction and verification of *MoIAD1* and *MoIAD2* deletion mutants; Table S1: Primers used in this study; Supplementary Dataset S1: Targeted metabolic analysis in this study.
