# Supplementary material for: Identification and Characterization of Auxin/IAA Biosynthesis Pathway in the Rice Blast Fungus Magnaporthe oryzae
Source: J Fungi (Basel). 2022 Feb 21;8(2):208. doi: 10.3390/jof8020208 (PMC8879529; doi:10.3390/jof8020208)
Supplement: Supplementary file 1 [file jof-08-00208-s001.zip › jof-1578312-supplementary.pdf]

Figure S1

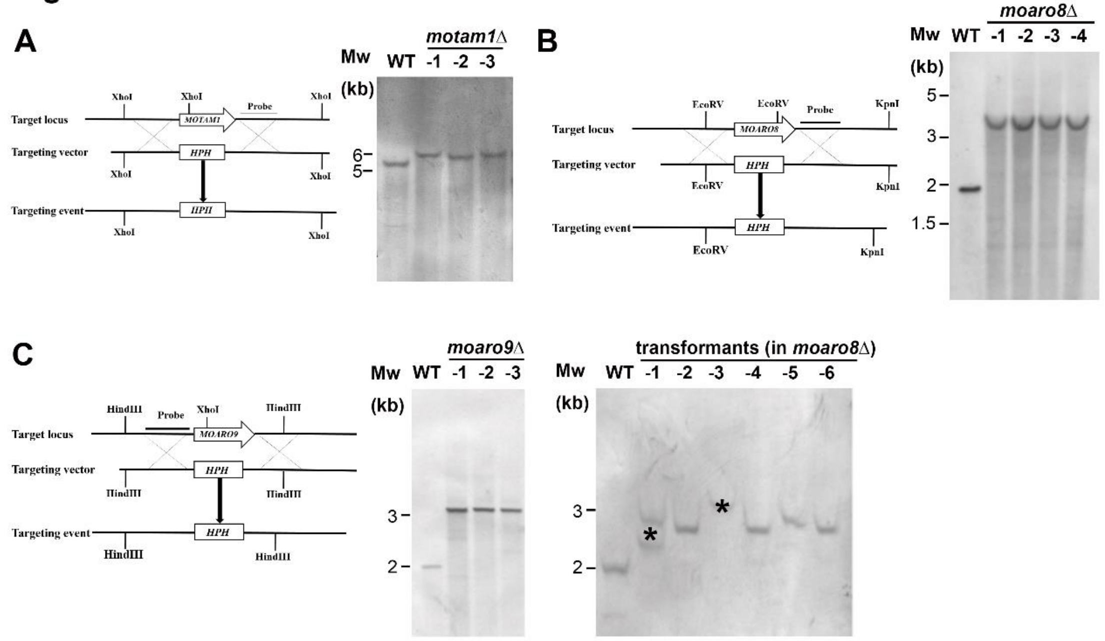

Figure S1. Construction and verification of deletion mutants.

(A-C) Schematic representation of gene deletion strategy and Southern blotting analysis for verification of gene deletion events, for *MoTAM1*, *MoARO8* and *MoARO9*. The targeted gene was replaced by hygromycin phosphotransferase cassette (*HPH*; for individual gene deletion), or by glufosinate ammonium resistance cassette (for deleting *MoARO9* in the *moaro8*Δ mutant background). The sites of the restriction enzyme used for digesting genomic DNA, and the probes for detecting the gene locus replacement, were labeled in the schemes, which were not drew to scale. For the *motam1*Δ mutants, the WT band of 5.5-kb was lack and concomitantly a single band of 6.6-kb was detected the *motam1*Δ mutants. For *MoARO8* gene, a single band of 1.6-kb was detected by probe in the WT strain, while disappeared in the moaro8Δ mutants. Instead, a single band of 3.3-kb was detected by the same probe. For single deletion of *MoARO9* gene, the WT band of 2.0-kb disappeared and a single band of 3.1-kb detected, diagnostic as the *moaro9*Δ mutants. The same probe detected a single band of 2.8-kb as diagnostic of *MoARO9* gene deletion in the *moaro8*Δ background. Asterisks denote transformants with random insertion but not targeted gene replacement.

Figure S2

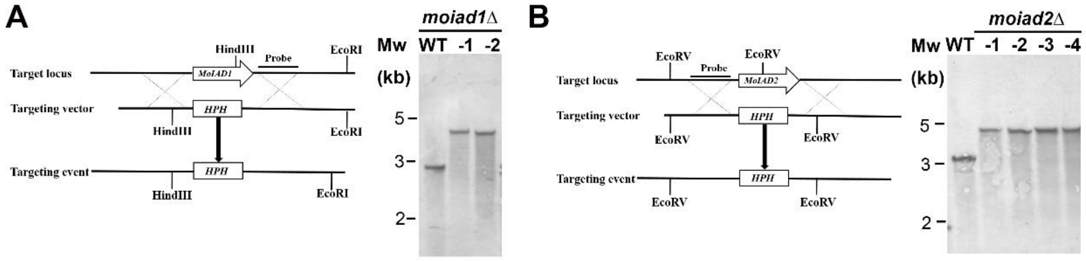

Figure S2. Construction and verification of *MoIAD1* and *MoIAD2* deletion mutants.

(A-B) Schematic representation of gene deletion strategy and Southern blotting analysis for verification of gene deletion events for *MoIAD1* and *MoIAD2*. The targeted gene was replaced by hygromycin phosphotransferase cassette (*HPH*). The sites of the restriction enzyme used for digesting genomic DNA of WT and deletion mutants were labeled. The schemes were not drew to scale. For *MoIAD1* gene, probe detected a single band of 2.8-kb in WT strain and a single band of 4.5-kb in the *moiad1*Δ mutants. For the *moiad2*Δ mutants, A single band of 3.0-kb was detected by probe in WT, while disappeared in the *moiad2*Δ mutants. Instead, a single band of 4.3-kb was detected by the same probe.

Table S1. Primers used in this study

Gene(Locus)	Description	Enzyme sites	Primer sequence
MoTAM1	Deletion construct	*Xho*I	UPF: 5'-GCCCTCGAGCTTGTCTAGTATAGTAGCCTTG-3'
(*MGG_14221*)		*BamH*I	UPR: 5'-GCCGGATCCTCCCGGTTTACTTCCCAGTG-3'
		*Pst*I	DSF: 5'-GCCCTGCAGTGTAGATTGTATATGCAAAAG-3'
		*Hind*III	DSR: 5'-GCCAAGCTTCTAGACAGCATACTCTTCCAG-3'
MoARO8	Deletion construct	*EcoR*I	UPF: 5'-CCGGAATTCCGCATAGTGTTGGTCTCAGA-3'
(*MGG_09919*)		*BamH*I	UPR: 5'-CGCGGATCCGATGAGTTGTGCGAACAGAG-3'
		*Pst*I	DSF: 5'-AACTGCAGGGTGGTCTTTCTCGGTTGT-3'
		*Hind*III	DSR: 5'-CCCAAGCTT GCGATGGTGTTATTGTTGC-3'
MoARO9	Deletion construct	*BamH*I	UPF: 5'-CGCGGATCCGAGATACCACCAGTTCCGTC-3'
(*MGG_08189*)	(in wild-type background)	*Xba*I	UPR: 5'-TGCTCTAGAGCTGGTGACGATTACAAATG-3'
		*Pst*I	DSF: 5'-AACTGCAGGTGAATAAGCGGCGTTTG-3'
		*Pvu*II	DSR: 5'-CCAGCTGGCCAGAAGTGAGTAGCGACT-3'
MoARO9	Deletion construct	*Kpn*I	UPF: 5'-GGGGTACCGAGATACCACCAGTTCCGTC-3'
(*MGG_08189*)	(in *moaro8*Δ background)	*Xba*I	UPR: 5'-TGCTCTAGAGCTGGTGACGATTACAAATG-3'
		*Sal*I	DSF: 5'-GCGTCGACGTGAATAAGCGGCGTTTG-3'
		*Pst*I	DSR: 5'-AACTGCAGGCCAGAAGTGAGTAGCGACT-3'
MoIAD1	Deletion construct	*EcoR*I	UPF: 5'-TATGGAGAAACTCGAGAATTCGCAAACAC ACAAATCGGGTCA-3'
(*MGG_03900*)		BamHI	UPR: 5'-GACTCTAGAACTAGTGGATCCAGGTGGAAGG TCGTGAGTTTGG-3'
		*Sal*I	DSF: 5'-ACGCGTCGACGGAGCCAGCAAAGAAATG-3'
		*Hind*III	DSR: 5'-CCCAAGCTTCAATCACAACCAAACTCAGC-3'

Table S1. *Cont*.

Gene(Locus)	Description	Enzyme sites	Primer sequence
MoIAD2 (*MGG_05008*)	Deletion construct	*Eco*RI	UPF: 5'-CCGGAATTCGAGACGGTAGCGAATGAGAG-3'
		*Xba*I	UPR: 5'-GCTCTAGAGTTACGGTAGTATGACGAGGG-3'
		*Hind*III	DSF: 5'-CCCAAGCTTGCTTGCTTTGGTTGACAGA-3'
		*Pvu*II	DSR: 5'-CCAGCTGGCTCAGACCCGAATAATCTC-3'
